# Biomarkers in the Diagnosis and Prognosis of Sarcoidosis: Current Use and Future Prospects

**DOI:** 10.3389/fimmu.2020.01443

**Published:** 2020-07-14

**Authors:** Raisa Kraaijvanger, Montse Janssen Bonás, Adriane D. M. Vorselaars, Marcel Veltkamp

**Affiliations:** ^1^Department of Pulmonology, ILD Center of Excellence, St. Antonius Hospital, Nieuwegein, Netherlands; ^2^Department of Pulmonology, University Medical Center, Utrecht, Netherlands

**Keywords:** biomarkers, serum, bronchoalveolar lavage, imaging biomarkers, future biomarkers, sarcoidosis

## Abstract

Sarcoidosis is a heterogeneous disease in terms of presentation, duration, and severity. Due to this heterogeneity, it is difficult to align treatment decisions. Biomarkers have proved to be useful for the diagnosis and prognosis of many diseases, and over the years, many biomarkers have been proposed to facilitate diagnosis, prognosis, and treatment decisions. Unfortunately, the ideal biomarker for sarcoidosis has not yet been discovered. The most commonly used biomarkers are serum and bronchoalveolar lavage biomarkers, but these lack the necessary specificity and sensitivity. In sarcoidosis, therefore, a combination of these biomarkers is often used to establish a proper diagnosis or detect possible progression. Other potential biomarkers include imaging tools and cell signaling pathways. Fluor-18-deoxyglucose positron emission tomography and high-resolution computed tomography have been proven to be more sensitive for the diagnosis and prognosis of both pulmonary and cardiac sarcoidosis than the serum biomarkers ACE and sIL-2R. There is an upcoming role for exploration of signaling pathways in sarcoidosis pathogenesis. The JAK/STAT and mTOR pathways in particular have been investigated because of their role in granuloma formation. The activation of these signaling pathways also proved to be a specific biomarker for the prognosis of sarcoidosis. Furthermore, both imaging and cell signaling biomarkers also enable patients who might benefit from a particular type of treatment to be distinguished from those who will not. In conclusion, the diagnostic and prognostic path of sarcoidosis involves many different types of existing and new biomarker. Research addressing biomarkers and disease pathology is ongoing in order to find the ideal sensitive and specific biomarker for this disease.

## Introduction

Sarcoidosis is a systemic inflammatory disorder of unknown cause which can lead to a variety of clinical symptoms. It commonly affects the lungs and intrathoracic lymph nodes, and is characterized by the formation of non-caseating epithelioid cell granulomas (1). In case of pulmonary involvement, granuloma formation can result in a decreased lung volume and diffusing capacity, with further shortness of breath (2). In most cases, the inflammation of sarcoidosis resolves within 2–3 years. In about 10–30% of the patients, however, the inflammation persists, leading to a chronic, sometimes progressive and even fibrotic disease for which treatment is required (3, 4). Mortality attributable to sarcoidosis is estimated between 0.5 and 5%, which is clinically relevant for a disease affecting relatively young people (5).

The diagnostic trajectory of sarcoidosis is long and complicated; it requires invasive methods like bronchoalveolar lavage and evidence of granuloma in lung or other tissue through a biopsy (6, 7). The diagnosis of sarcoidosis is then established by excluding other diseases with similar clinical or histopathological features (8).

The discovery of a specific biomarker for this disease would help diagnose sarcoidosis. According to the National Institutes of Health Biomarker Definitions Working Group, a biomarker is “a characteristic that can be objectively measured and evaluated as an indicator of normal biological processes, pathogenic processes, or pharmacologic responses to a therapeutic intervention” (9). Such predicative biomarkers are already widely used to detect the presence and severity of a variety of inflammatory diseases. However, no specific biomarker for sarcoidosis has so far been identified (2). A predictive biomarker would also be useful for treatment decisions. Because of the heterogeneity of sarcoidosis, it currently remains unclear who will benefit from a specific type of treatment (10).

The ideal biomarker should not be related to other diseases and should be highly sensitive. Moreover, it should not be invasive, and should be reproducible. Also, the ideal biomarker should be inexpensive (11). A combination of measurable biomarkers and cytology would help to understand the changes within the body that are caused by sarcoidosis, as well as the occurrence and progression of the disease, and would enable personalized treatment (2).

In this review, we address current and possible future biomarkers in sarcoidosis. Serum and bronchoalveolar lavage biomarkers are discussed in terms of antigen presentation, granuloma formation, and T-cell activation. As regards imaging, we focus on the value of chest X-ray, fluor-18-deoxyglucose positron emission tomography (^18^F-FDG PET) and high-resolution computed tomography (HRCT) as diagnostic and prognostic tools in sarcoidosis.

## Serum Biomarkers

**Figure 1:**
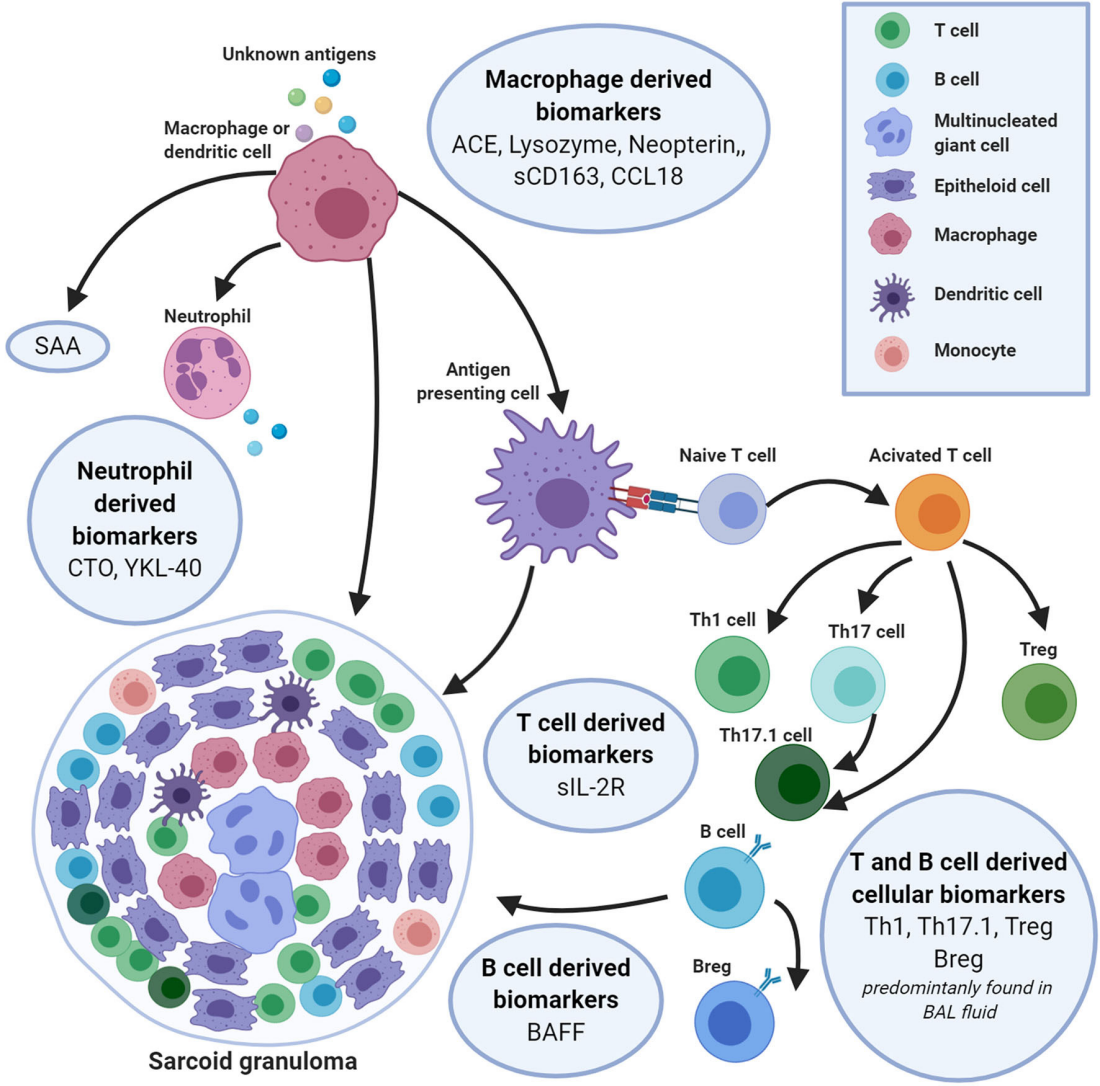
An integrated overview of serum and bronchoalveolar lavage fluid biomarkers produced by cells of the innate and adaptive immune system, involved in the formation of granulomas in sarcoidosis. The sarcoidosis granuloma consists of a tightly formed core of epithelioid and multinucleated-giant cells (MGCs) encircled especially by T helper (Th) cells, but also by B cells, macrophages and dendritic cells (DCs). During this granuloma formation a variety of biomarkers is released by these inflammatory cells. Macrophages are key players in granuloma formation and produce a number of inflammatory biomarkers [e.g., serum angiotensin-converting enzyme (sACE), lysozyme, neopterin, CD163, C-C motive chemokine ligand 18 (CCL18), serum amyloid A (SAA)]. Macrophages activate T-cells by presenting a triggering antigen, which results in an upregulated expression of soluble interleukin 2 receptor (sIL-2R). Apart from T-cells, B-cells also have shown to play a role in granuloma formation. Crucial for the B-cell maturation and function is the biomarker B-cell activating factor (BAFF). All these biomarkers may be useful for the diagnosis and prognosis of sarcoidosis. Figure created with Biorender.com.

Most serum biomarkers found in sarcoidosis are produced by inflammatory cells involved in granuloma formation (Figure 1). In this overview, biomarkers are discussed for each of the cell types associated with the biomarkers (Table S1).

### Macrophages

Monocyte-derived macrophages are known to be key players in granuloma formation. In order to regulate granuloma formation, monocytes, and macrophages produce a number of cytokines, chemokines and other signaling proteins. Many of these have been previously described as potential serum biomarkers in sarcoidosis and normally correlate with granuloma burden (12).

#### Serum Angiotensin-Converting Enzyme

The most well-known serum biomarker in sarcoidosis is serum angiotensin-converting enzyme (sACE). sACE is an acid glycoprotein which converts angiotensin I into angiotensin II. It is mainly produced by activated alveolar macrophages and correlates with granuloma burden and radiological stages II and III (13). Elevated serum levels of ACE have been intensively studied since 1975 and this is currently the most frequently used laboratory test in sarcoidosis (14). Roughly 30–80% of sarcoidosis patients have increased sACE levels, and sensitivity ranges between 22 and 86% and specificity between 54 and 95% (8). Due to the low sensitivity of sACE levels, their value as a diagnostic or prognostic tool remains a matter of debate. The low sensitivity of elevated sACE levels in sarcoidosis patients is partly due to the fact that sACE levels have been found to be increased in several other inflammatory diseases, like tuberculosis, berylliosis, histoplasmosis, Gaucher's disease and many others (15–18).

Furthermore, ACE levels in the normal range could in fact be elevated in specific patients. This is based on the influence of an insertion (I) or deletion (D) polymorphism in the ACE gene on sACE levels (19). In healthy controls, subjects with an II genotype had a significantly lower serum ACE concentration compared to subjects with a DD genotype. In clinical practice, a Z-score has been developed that corrects the ACE activity for the I/D polymorphism (19, 20). Using the correction for this I/D polymorphism results in a different interpretation in 8.5% of measurements (19).

ACE could also have a role in predicting treatment outcome. High serum levels of ACE before treatment correlate significantly with lung function improvement after 6 months of methotrexate treatment (21). What has to be kept in mind is the use of ACE inhibitors in patients with sarcoidosis (22). If patients with sarcoidosis use ACE inhibitors, serum ACE levels cannot be used in diagnosis or disease monitoring, and should be interpreted carefully (23).

#### Lysozyme

Lysozyme is a bacteriolytic enzyme that hydrolyses glycosidic bonds in order to degrade peptidoglycans in bacterial cell walls. At the site of infection lysozyme activity limits the cause of inflammation by rapidly degrading peptidoglycans (24). In sarcoidosis, lysozyme is produced by monocyte-macrophage systems and epithelioid cells, and is involved in granuloma formation. Increased concentration of lysozyme is mainly observed at onset of disease and has low sensitivity for sarcoidosis. Hence, lysozyme is more suitable as a prognostic rather than a diagnostic tool (25, 26).

#### Neopterin

Neopterin is a non-specific marker of inflammation produced by activated monocytes, macrophages, dendritic cells, and endothelial cells upon stimulation mainly by interferon gamma (IFN-γ) (27). Neopterin is released in response to cytokines mostly produced by T-cells and natural killer cells. The interaction between T-cells, macrophages, and dendritic cells in the process of granuloma formation could be due to the higher neopterin levels which have been found in sarcoidosis patients with active disease (28, 29). Although serum neopterin levels seem to be increased in sarcoidosis patients, the specificity of neopterin for sarcoidosis is low; therefore, it has little value as a diagnostic biomarker. Nevertheless, it could be a potential marker for disease activity and predictor of progression, but further research is required (30).

#### YKL40

The human cartilage glycoprotein-39 or YKL-40 is a growth factor for fibroblasts and vascular endothelial cells and is secreted by macrophages and neutrophils. Increased serum YKL-40 levels have been found in patients with diseases characterized by inflammation, tissue remodeling and ongoing fibrosis (31). In sarcoidosis patients, serum YKL-40 levels have been found to be elevated and to inversely correlate with diffusing capacity of the lung for carbon monoxide (DLCO) at presentation. In addition, serum YKL-40 levels are higher in patients with active sarcoidosis than in patients with inactive sarcoidosis (32, 33). A correlation has been found between YKL-40 levels and both sIL-2R levels and sACE levels in patients with active sarcoidosis, suggesting YKL-40 to be a marker for granuloma burden (33). At this moment, however, little is known about the value of serum YKL-40 levels, and more research is required to determine the value of YKL-40 as a diagnostic or prognostic biomarker in sarcoidosis (28).

#### sCD163

CD163 is a transmembrane hemoglobin-haptoglobin scavenger receptor expressed selectively on most macrophages in human tissues and on at least 10–30% of monocytes. Expression of CD163 is increased by IL-6, IL-10, glucocorticoids, and most immunomodulatory factors. Expression of CD163 decreases under the influence of tumor necrosis factor (TNF), interferon gamma (IFN-γ), and transforming growth factor beta (TGF-β). These are all cytokines involved in granuloma formation and disease activity. This may suggest a correlation between the expression of CD163 and inflammatory status in sarcoidosis patients. Shortly after activation of the toll-like receptors-2,4, and 5 (TLR), CD163 is shed into the environment as soluble CD163 (sCD163) (34). This quick release of sCD163 into the environment after TLR activation implies a role in inflammation, but no specific function of sCD163 has yet been identified (35). sCD163 may inhibit T-cell proliferation and activation, although the exact mechanism for this effect remains unclear. Nevertheless, the increase of sCD163 levels after stimulation of TLR suggests that sCD163 may play a role in inflammatory disorders mediated by monocyte-macrophage lineage cells. In sarcoidosis patients sCD163 was found to be significantly increased in comparison to healthy controls and correlated with sIL-2R and sACE levels (36). Although little is known about the role of sCD163 in sarcoidosis, it may be a useful biomarker with potentially high sensitivity but low specificity. In other diseases like RA, MS and Crohn's disease, sCD163 has been associated with disease activity and suggested as a useful predictor of progression (35, 37).

#### CC Chemokine Ligand 18

C-C motive chemokine ligand 18 (CCL18) is a CC chemokine produced primarily by antigen-presenting cells such as macrophages, dendritic cells, and peripheral blood monocytes. CCL18 is chemotactic for both naive and activated T-lymphocytes. Elevated levels of CCL18 have been detected in serum of patients with T-cell helper 2 (Th2) predominant diseases like idiopathic pulmonary fibrosis, bronchial asthma and scleroderma (38). CCL18 can stimulate the mRNA and protein production of collagen, possibly stimulating fibrosis. In sarcoidosis patients, elevated levels of CCL18 have been found in patients with active disease (39). Unfortunately, CCL18 is elevated in most interstitial lung diseases, and also in Gaucher's disease, so it is not suitable as a diagnostic biomarker for sarcoidosis. However, CCL18 has potential to be used as a marker to monitor disease and predict progression (39), as was already suggested by Prasse et al. (40), who demonstrated that CCL18 levels in bronchoalveolar lavage (BAL) correlate with the scadding stage.

#### Serum Amyloid A

Serum amyloid A (SAA) is produced by the liver during an acute phase reaction. In inflammatory conditions, macrophages produce high levels of SAA, which indicates that elevated levels of SAA are a clinical marker of inflammation. Elevated levels of SAA are found in several inflammatory diseases like RA and Crohn's disease (41). Elevated levels of SAA have also been found in sarcoidosis, and appear to correlate with a decline in lung function (42). Sarcoidosis has been suggested as a heterogeneous disease, with multiple potential triggers such as metals or micro-organisms (43). If in the future patients could be distinguished based on possible triggers, it would be interesting to study SAA as a biomarker in a specific subgroup of sarcoidosis patients with micro-organisms such as mycobacteria or propionic bacteria as suspected triggers (44).

#### Chitotriosidase

Chitotriosidase (CTO) is an enzyme of the chitinase family. It degrades chitin, a polymer found in cell walls of fungi and the exoskeletons of insects and crustaceans. Pulmonary neutrophils and macrophages can secrete CTO upon stimulation of toll-like receptors (TLRs) by IFN-γ, TNF, and granulocyte/macrophage colony-stimulating factor (GM-CSF). CTO serum levels directly correlate with sACE levels (45). Highest serum levels were found in patients with progressive disease and were found to decrease upon treatment with prednisone or other immunosuppressant therapy. As a diagnostic marker, CTO is less useful due to low specificity. CTO serum levels have been found to be elevated in other diseases like Gaucher's disease, malaria, multiple sclerosis, atherosclerosis, Alzheimer's disease, and tuberculosis (20). However, CTO sensitivity and specificity are higher than those of other serum biomarkers, making CTO a potentially useful biomarker in sarcoidosis. CTO serum levels have prognostic value in sarcoidosis and can be used to monitor disease activity, with the potential to be used as a diagnostic tool for this disease (45, 46).

### Monocytes

As mentioned above, macrophages are key players in granuloma formation. However, macrophages are difficult to study or use as diagnostic/prognostic biomarkers, since they are predominantly found in tissue and granuloma. Monocytes are precursors of macrophages and are found in the bloodstream, making them accessible and interesting as biomarkers in sarcoidosis. There are three subtypes of monocytes, classical monocytes (CD14++/CD16–), intermediate monocytes (CD14+/CD16+) and non-classical monocytes (CD14–/CD16++). Intermediate and non-classical monocytes are more inflammatory type monocytes, and have been found to be elevated in sarcoidosis (47). In addition, treatments with prednisone and infliximab have been shown to have a downregulating effect on intermediate and non-classical monocytes, suggesting a role in disease activity and prognostic value for response to treatment (48, 49). In addition to the CD14 and CD16 surface markers, many different monocyte surface markers have been studied, with promising results (50, 51). However, circulating intermediate and non-classical monocytes have also been found to be elevated in other diseases, like cardiovascular diseases and other interstitial diseases, thus decreasing its specificity (52, 53). Circulating subtypes of monocytes are potentially interesting prognostic biomarkers, but further research is required.

### T-Cells

T-cells play an important role in the development of granulomas. Antigen presenting cells like macrophages present peptides to T-helper (Th) cells via MHC class II molecules. This activates T-cells and leads to greater proliferation and recruitment of neutrophils and monocytes (54). Based on T-cell activation, many T-cell cytokines and chemokines have been described as potential biomarkers for sarcoidosis. The activation of T-cells can be seen as a crucial step in the perpetuation of granuloma formation.

#### Serum Soluble Interleukin 2 Receptor

Serum soluble interleukin 2 receptor (sIL-2R) is the circulating form of the membrane IL-2R, a proposed marker of disease activity in sarcoidosis. Upon activation, Th1 cells upregulate the expression of IL-2R on the cell surface, and are able to shed sIL-2R into circulation (55). Increased sIL-2R levels are therefore considered to be a marker of Th1 cell activation in the formation and perpetuation of granuloma (56). Increased levels of sIL-2R in sarcoidosis patients have been described since 1983 (57). Unfortunately, elevated sIL-2R levels are not specific for sarcoidosis, as elevated serum levels of sIL-2R are found in other granulomatous diseases, hematological malignancies, and various autoimmune disorders (10). Even though the use of sIL-2R as a diagnostic marker for sarcoidosis remains a matter of debate, a recent study performed in patients suspected of sarcoidosis has shown a sensitivity of 88% and a specificity of 85% (58). This indicates that sIL-2R can be a useful tool in the diagnosis of sarcoidosis when combined with other (imaging) biomarkers and clinical features in the process of diagnosis.

sIL-2R seems to correlate with active disease and multiple organ involvement, and can possibly predict progression and relapse after discontinuation of therapy (59–61) Furthermore, sIL-2R can be used as a prognostic tool to determine if therapy is needed and/or to predict relapse after discontinuation of therapy (21, 59). Moreover, sIL-2R can be used in serial measurements during therapy or in follow-up to evaluate treatment effect (11).

It is important to state that patients with impaired renal function have elevated sIL-2R. This can result in high sIL-2R levels in the absence of active disease. Since renal impairment can occur in sarcoidosis patients, this effect has to be kept in mind when sIL-2R is used as a marker to monitor disease activity (59).

### B-Cells

Although the innate immune system and T-cell immunity are known to be involved in the pathogenesis of sarcoidosis, it is becoming increasingly evident that B-cells are involved in granuloma formation as well. B-cell accumulation has been shown in granuloma and pulmonary lesions, and a positive effect of B-cell depletion has been reported (62, 63).

#### B-Cell Activating Factor

B-cell activating factor (BAFF) is a cytokine of the TNF family with a critical role in B-cell development and function. *In vivo* and *in vitro* studies have shown that blocking of BAFF results in reduced follicular and marginal zone B-cell numbers, while overexpression of BAFF in mice resulted in an increase in activated B-cells, activated T-cells, autoantibody production, and hypergammaglobulinemia. In sarcoidosis patients, higher levels of BAFF have been found in serum in comparison to healthy controls (64–66). However, elevated BAFF levels are not specific for sarcoidosis, as these have also been found in other immunomodulatory diseases like systemic lupus erythematosus (SLE) and rheumatoid arthritis (RA) (67). In addition to elevated serum levels being found in sarcoidosis patients, a correlation between BAFF levels and disease severity has been shown. Highest levels of BAFF in sarcoidosis patients are associated with multiple organ involvement, decline in pulmonary function, and more advanced chest radiographic stages (II/III) (64). In a different study, higher serum levels of BAFF were associated with a higher frequency of eye and skin involvement and with increased levels of sACE, lysozyme, and IFN-γ (66). Although much is still unknown about the mechanisms of BAFF in sarcoidosis patients, results reported so far seem promising enough to further explore the value of BAFF as a prognostic or even diagnostic biomarker in sarcoidosis.

#### Naïve and Memory B-Cells

The B-cell ablative rituximab targets CD20, a marker expressed on the surface of naïve and memory B-cells, and has been shown to yield clinical improvement in some sarcoidosis patients (63, 68). This observation suggests a role for naïve and memory B-cells in sarcoidosis pathophysiology. When phenotyping B-cells, subpopulations are distributed differently in sarcoidosis patients in comparison to healthy controls (62, 69, 70). Naïve B-cell populations have been found to be elevated in sarcoidosis patients, and memory B-cells have been found to be downregulated in sarcoidosis patients (62). However, not all studies found the memory B-cells to be downregulated (69). When comparing sarcoidosis patients with and without pulmonary involvement, only the naïve mature B-cells are different between the two groups: sarcoidosis patients without pulmonary involvement do not have increased numbers of naïve mature B-cells.

Not only are numbers of naïve and memory B-cells altered, but sarcoid B-cells were found to be anergic in chronic sarcoidosis. This anergy could be partly due to the reduced levels of NF-κB/p65 found in sarcoid B-cells. B-cells with reduced NF-κB have an impaired response to antigens (69, 71). Furthermore, when memory B-cells are reduced, this may lead to a defective antibody response that is not able to eliminate the antigens responsible for granuloma formation. These findings suggest an important and possibly pathogenic role for B-cells in sarcoidosis, but the involvement of the different B-cell populations in granuloma formation remains unclear (62).

#### Regulatory B-Cells

Regulatory B-cells (Bregs) are IL-10 producing B-cells. This type of B-cell has been found to reduce inflammation through cytoplasmic IL-10 expression (72). Phenotyping B-cell populations in sarcoidosis patients revealed that circulating numbers of IL-10 producing B-cells were elevated in patients with active sarcoidosis. Both the frequency and absolute numbers were significantly higher in active sarcoidosis. Although current knowledge of IL-10 producing B-cells is insufficient to draw any conclusion on the role of Bregs, it seems that there is an altered B-cell homeostasis in active sarcoidosis.

This observation does appear to be in line with findings in other inflammatory diseases like SLE, RA, and multiple sclerosis. Furthermore, since IL-10 is important in the development of fibrosis, this association could justify further research into this population of B-cells (62, 65).

In conclusion, B-cells are relatively new and somewhat unknown cells in sarcoidosis. However, since B-cells seem to be involved in several processes in sarcoidosis pathogenesis, the expectation would be that BAFF levels and memory, naïve, and regulatory B-cell counts could be used to assess disease activity and could possibly be used in the diagnosis of sarcoidosis. However, further research is required before B-cell counts and BAFF levels can be used as biomarkers in sarcoidosis.

### Combining Serum Biomarkers

All biomarkers mentioned above as serum biomarkers appear to have the same limitations, namely, insufficient sensitivity and specificity. Since many biomarkers have shown a mutual positive correlation (25, 28, 73), different combinations are being explored. Using a combination of different biomarkers, various studies have already demonstrated an increase of both sensitivity and specificity (74–76).

## Bronchoalveolar Lavage Fluid

The diagnosis of sarcoidosis is often confirmed by analyzing bronchoalveolar lavage fluid (BALF) (77). Various cells and soluble components in BALF, such as proteins and cytokines, have been addressed in different immunological studies. These studies have considerably increased our understanding of the immunopathogenesis of sarcoidosis (29, 77) (Table S2).

### CD4/CD8 Ratio

In sarcoidosis, the percentage of lymphocytes in BALF is often increased. Sensitivity of lymphocytosis was found to range between 68 and 95% (78, 79). This variation is related to the fact that an increase in lymphocytes in BALF is nonspecific for sarcoidosis, as it is also observed in other granulomatous and lung diseases (10, 29, 80). The observation that CD4+ T-cells play an important role in the development of sarcoidosis has helped distinguish sarcoidosis from other diseases (81). Measurement of the ratio of CD4/CD8 T-cells has been used to differentiate sarcoidosis from other diseases since the 1980s (29, 80). Increased CD4/CD8 ratio was observed in sarcoidosis patients, with a sensitivity of between 54 and 80% and a specificity of between 59 and 80% (10, 29, 78, 79). In the clinic, the diagnosis of sarcoidosis is supported by a CD4/CD8 ratio >3.5 and lymphocytosis > 15%. However, BALF lymphocytosis is not a universal finding in sarcoidosis (81). A recent study by Darlington et al. (82) showed that the measurement of the proportion of T-cell receptor (TCR) CD4+ Va2.3+ T-cells in BALF could also be a biomarker in addition to the CD4/CD8 ratio, to support a sarcoidosis diagnosis. TCR Va2.3 gene segments were found to be increased in BALF, especially in patients with an HLA-DRB1^*^03 genotype, which is usually found in patients with Löfgren's syndrome (LS). This biomarker therefore proved to be more useful for the diagnosis of LS, while the CD4/CD8 ratio is useful for the diagnosis of any type of sarcoidosis. On the other hand, specificity proved to be higher for CD4+ Va2.3+ T-cells compared to the CD4/CD8 ratio in BALF (97 vs. 92%), which will help with the diagnosis of sarcoidosis (82). Despite the frequent use of the CD4/CD8 ratio for the diagnosis of sarcoidosis, this ratio does not reflect the severity of the disease (29).

### CD103+CD4+/CD4+ Ratio

While the CD4/CD8 ratio is useful for the diagnosis of sarcoidosis, sensitivity and specificity of this biomarker are low. Therefore the expression of other cellular markers in this subset of T-cells have been explored.

Expression of the aEβ7/CD103β7 integrin (CD103) has been related to the retention of intraepithelial lymphocytes (IEL) in mucosal tissue of the lung. CD103 is expressed by lymphocytes within the bronchial epithelium, some alveolar wall lymphocytes and CD4+ T-cells in the BAL. Expression of CD103 on CD4+ T-cells in BAL fluid is found to be different in ILD patients, depending on the type of disease (83). In patients with sarcoidosis, numbers of CD103 CD4+ T-cells in BAL fluid are significantly lower than in other lung diseases (83). Therefore, differential expression of CD103 on CD4+ T-cells could serve as a diagnostic marker for sarcoidosis. However, there is some controversy on the use of the CD103+CD4+/CD4+ ratio as a diagnostic tool for sarcoidosis. The sensitivity and specificity seems to depend strongly on the population used in the study. Heron et al. (84) and Bretagne et al. (85) show a significant decrease in the CD103+CD4+/CD4+ ratio in sarcoidosis patients in comparison to other ILD while a study by Hyldgaard et al. (79) does not show a similar decrease. The difference in outcome might be explained by the inclusion of patients without alveolar lymphocytosis by Hyldgaard et al. (79).

While the CD103+CD4+/CD4+ appears to be a useful diagnostic tool in sarcoidosis caution should be taken with the interpretation of this ratio. Therefore, a CD103+CD4+/CD4+ ratio 0.2 can be seen as additional evidence pointing towards a diagnosis of sarcoidosis.

### T-Helper 17.1 Cells

For a long time, sarcoidosis was seen as a Th1-driven disease, because of the variety of type 1 specific cytokines present in BALF and serum of sarcoidosis patients, including IFN-γ, IL-12, and IL-18. However, nowadays it is accepted that sarcoidosis is driven by a combination of Th1- and Th17-associated factors (86). Th17 cells normally produce IL-17A and IL-17F, but in sarcoidosis Th17 cells were also observed to produce IFN-γ (12, 87). These cells are referred to as Th17.1 cells and are thought to derive from classically polarized Th17 cells. Th17 cells have a high plasticity and were found to be able to differentiate into a Th1-like phenotype, in which the majority mainly produce IFN-γ (87, 88). Both Th17 and Th17.1 cells were identified in sarcoidosis patients, and these Th-cells were found to express different chemokine receptors, and demonstrated different cytokine effector functions. The number of Th17.1 cells proved to be significantly higher in the BALF, but not the blood, of sarcoidosis patients, compared to healthy controls (88). Th17.1 cells are promising immunological markers in the diagnosis of sarcoidosis, but results are based on small cohorts, so further research is required to determine the diagnostic value. Additionally, it was seen that there is a significant association between the number of Th17.1 cells in BALF and the development of chronic sarcoidosis, making these cells an interesting prognostic marker for the development of (chronic) pulmonary sarcoidosis (87).

### Regulatory T-Cells

Regulatory T-cells or Treg cells may also play an important role in the development of sarcoidosis. Treg cells normally suppress immune responses by the host immune system against self and foreign antigens. In the case of sarcoidosis, it is hypothesized that Tregs are functionally defective or exhausted, making them unable to fully suppress the immune responses (89). The dysfunction of Tregs in sarcoidosis, resulting in impaired self-tolerance and failed immune homeostasis, could be an important element in the pathogenesis of sarcoidosis (90). A Polish study showed that the dysfunction of Tregs resulted in an exaggerated Th1 response in sarcoidosis patients (91). The diagnostic value of Tregs in sarcoidosis remains to be determined. A number of possible correlations between Tregs and disease activity have been described, with prognostic values. A Scandinavian study showed that a low Treg level in BALF was associated with a favorable prognosis (92). On the other hand, a German study reported that a decreased Treg level in BALF corresponded with the development of chronic disease (93).

Although there are thus conflicting results on the presence and effect of Tregs in active sarcoidosis, what is clear is that an imbalance exists between Th17 and Tregs. Th17 cells are elevated in the blood of sarcoidosis patients, while the number of Tregs is decreased (90). The Treg/Th17 ratio proved to be inversely related to disease activity, decreasing in those with relapsing pulmonary sarcoidosis and increasing in those with chronic active disease (12, 94).

### Neutrophils

Neutrophils are important players in the innate immune system, but their role in sarcoidosis is still little understood. Neutrophils are attracted by chemokines, such as IL-8, which are released by monocytes and macrophages (12). Patients in an advanced radiological stage of sarcoidosis (stage II or III), or with an unfavorable evolution, were reported to have an elevated percentage of neutrophils in BALF compared to healthy controls. Furthermore, BALF neutrophil levels were significantly higher in patients with progressive disease compared to those with stable disease. Therefore, neutrophils may be useful markers of progressive disease (29, 95). A possible origin of this correlation could be the large number of proteases, like collagenase or gelatinase, which may initiate collagen destruction and remodeling, leading to the development of pulmonary fibrosis (12).

### Natural Killer (NK) Cells

Other immune cells in BALF which are associated with poor outcome and an advanced radiological stage are natural killer (NK) cells (95). NK cells (CD3–CD16/56+) are part of the first-line defense of the immune system (96). They are suspected to play a role in several inflammatory pulmonary diseases because of their ability to produce cytokines (95). IFN-γ is released by active NK cells and stimulates TNF-α secretion from alveolar macrophages, which is associated with progressive and corticosteroid resistant disease (95). An impaired lung function was shown to be associated with a high level of NK cells in the lungs. Furthermore, sarcoidosis patients requiring steroid treatment also had a higher percentage of these cells in BALF (95).

### Natural Killer T (NKT) Cells

Natural killer T (NKT) cells (CD3+CD16/56+) are a regulatory T cell lineage which both express T cell (CD3) and NK (CD16/56+) receptors on their surface membranes, thereby influencing both Th1 and Th2 cytokine systems and stimulating cell-mediated immunity or suppressing autoimmune responses (96, 97). NK cells are able to release a various cytokines determined by antigen signal strength, including IFN-γ, TNF-α, and a variety of interleukins (IL-4, IL-10, IL-13, IL-17, IL-21) (97). In sarcoidosis patients reduced numbers of NKT cells were found in blood and BALF (98–100). However, there are also reports on the accumulation of NKT cell in granulomatous lesions of sarcoidosis patients (99). Deficiency and/or impaired function of NKT cells results in loss of the immunoregulation, which might contribute to the prolonged T-cell activity characteristic for sarcoidosis (101).

### CXCL9, CXCL10, and CXCL11

The monocyte-macrophage cell lineage is the origin of the expression of a variety of cytokines and chemokines that play many different roles in the inflammatory phenotype of sarcoidosis, including T-cell attraction and promotion of Th1/Th17 differentiation (8). Some interesting chemokines were found to be elevated in the BALF and serum of sarcoidosis patients. These included chemokines that are part of the CXC chemokine subfamily; CXCL9, CXCL10, and CXCL11 (102, 103). These chemokines bind to the CXCR3 receptor and thereby recruit CD4+ T-cells, monocytes and other inflammatory cells to the site of inflammation (103). Although these chemokines share common functions, there are differences in their biological properties (103, 104). One major difference is the fact that CXCL9 and CXCL11 can solely be induced by IFN-γ, while CXCL10 can also be induced by TNF-α, IFN-α, and LPS (103, 104). These differences may influence the clinical outcomes of these chemokines (104). In tuberculosis, this chemokine subfamily was already found to represent useful biomarkers for the prediction of disease progression and therapy response (105, 106).

In the case of sarcoidosis, a recent study by Arger et al. (103) showed that CXCL10 correlates negatively with lung function, and that elevated CXCL10 also correlates with higher dyspnea scores in longitudinal analyses. These correlations could not be found for CXCL9. However, CXCL9 proved to be positively associated with the total number of organs involved in sarcoidosis. CXCL11 proved to be negatively associated lung function and correlated positively with the number of organs involved (103). It was speculated that each of these chemokines could be used to help predict the clinical outcome of a sarcoidosis patient, as well as acting as biomarkers for both pulmonary outcomes and response to therapy (103).

### Krebs von den Lungen-6

Krebs von den Lungen-6 (KL6) is a human high molecular weight MUC1 mucin protein derived from type II pneumocytes and respiratory bronchiolar epithelial cells (107). An increased level of KL-6 reflects damaged or regenerating type II pneumocytes (108).

Elevated levels of KL-6 have been found in BALF and serum in patients with idiopathic pulmonary fibrosis, sarcoidosis, and other ILDs (109–112).

The fact that it is not a disease specific marker indicates that it has no major role in diagnosing sarcoidosis. However, in sarcoidosis patients both serum and BALF levels of KL-6 correlate with higher serum ACE levels and CD4+/CD8+ ratio (25, 112). Suggesting usefulness as a disease monitoring tool. It is demonstrated that the highest level of KL-6 can be found in patients with radiological stage IV pulmonary sarcoidosis (25, 76, 111) suggesting a potential role as a marker of severity of pulmonary sarcoidosis.

### Combining BALF Biomarkers

The biomarkers mentioned above give limited information about pulmonary sarcoidosis as individual biomarkers. However, combining some of these biomarkers may be helpful in the diagnosis and prognosis of pulmonary sarcoidosis. CD4/CD8 ratio and CD103+CD4+/CD4+ ratio appear to be useful diagnostic biomarkers to support the diagnosis of sarcoidosis, while disease severity can be predicted and monitored by a correlation between KL-6 and CD4+/CD8+ ratio. Furthermore, disease progression can be monitored trough the percentage of neutrophils, NK cells, and NKT cells in BALF. In addition, Th17.1 cells and Treg cells are promising prognostic biomarkers, however, further research is required to determine the diagnostic value. The BALF chemokines CXCL9-11 in their turn may be useful biomarkers for treatment decisions.

## Imaging Biomarkers

Clinical manifestations of sarcoidosis are highly variable and any organ can be affected. For pulmonary sarcoidosis, chest X-ray, and high-resolution computed tomography (HRCT) are frequently used. However, in sarcoidosis patients with extrapulmonary manifestations, such as cardiac sarcoidosis or neurosarcoidosis, other imaging modalities are warranted. For these patients, magnetic resonance imaging (MRI) and fluor-18-deoxyglucose positron emission tomography (^18^F-FDG PET) scanning are increasingly recognized as essential imaging techniques for adequately identifying sarcoidosis localization and determining disease management (113).

### Chest X-ray

Conventional chest radiography is performed in most patients with sarcoidosis and is abnormal in over 90% of cases (114). Sarcoidosis is commonly staged according to its appearance on chest X-ray. This staging system was introduced by Scadding almost six decades ago (115), but still holds a prominent position in assessment of sarcoidosis. Scadding classified chest radiography findings in sarcoidosis into five stages, shown in Table 1.

**Table 1:** Radiographic staging of sarcoidosis patients at presentation according to the scadding criteria.

**Radiographic stage**	**Chest X-ray**	**Frequency (%)**	**Resolution (%)**
0	Normal	5–15	
I	BHL	25–65	60–90
II	BHL and pulmonary infiltrates	20–40	40–70
III	Pulmonary infiltrates without BHL	10–15	10–20
1V	Advanced pulmonary fibrosis	5	0

*The estimated frequency at presentation is given as well as the probability of spontaneous resolution during disease course (BHL, bilateral hilar lymphadenopathy) (1, 114, 116)*.

An interesting feature of the abovementioned Scadding stages is the fact that it gives prognostic information both on resolution of disease as well as mortality (1, 114, 116, 117). Spontaneous resolution varies from 60 to 90% in stage I disease to no resolution in stage IV fibrotic disease. Furthermore, Kirkil et al. (117) demonstrated that mortality rates were correlated to Scadding stages with the highest mortality in patients presenting with stage IV disease.

Shortcomings of conventional chest X-ray is the fact that in absence of pathological confirmation, clinical features together with chest X-ray can be diagnostic in stage I and II disease but are less accurate in stage 0, II, and IV disease (1). Furthermore, only moderate agreement on Scadding stages is reported in literature (118). Finally, Judson et al. (119) demonstrated that chest X-ray was inadequate to reliably detect acute exacerbations of pulmonary sarcoidosis. These are some reasons why the HRCT now has a prominent place in the diagnosis and monitoring of sarcoidosis.

### High-Resolution Computed Tomography

HRCT has a solid position in the diagnostic approach and follow-up of sarcoidosis, as it provides high-resolution slides of the pulmonary parenchyma. Although widely used, quantification of HRCT results remains a difficult task even for experienced radiologists and pulmonologists. A reliable inter- and intra-patient comparison of results requires standardized quantification of HRCT findings.

In a pioneering approach to objectively quantifying HRCT results, Oberstein et al. developed a scoring system based on the sum of typical patterns of parenchymal involvement, combined with lymph node enlargement and pleural involvement. The Oberstein score yielded high correlation coefficients with BAL total cell count and sIL-2R (120).

Drent et al., who also found a good interobserver agreement in a retrospective cohort of 80 sarcoidosis patients, later replicated the Oberstein score. They also found a correlation between pulmonary function and HRCT abnormality scores for all subgroups except lymph node enlargement (121). When used together with serum sIL-2R values, the HRCT scoring system can predict disease activity and could replace ^18^F-FDG PET (122). Although this finding was not replicated, it could be an important finding especially with regard to cost-effectiveness and the fact that ^18^F-FDG PET is not universally available.

In order to objectively quantify HRCT results regarding the extent and activity of sarcoidosis, Benamore et al. more recently developed the CT activity score (CTAS), which consists of CT extent scores for nodularity, ground-glass opacification, interlobular septal thickening and consolidation. The total CTAS was found to predict response to treatment in FVC after 1 year in their cohort of 100 patients. Furthermore, CTAS had a high degree of interobserver agreement (123).

A retrospective study of a cohort of 57 patient reproduced these results and found significant correlations with lung function changes after six months of therapy (Change in %VC: *r* = 0.543, *P* < 0.001). Furthermore, higher CTAS scores were seen in patients with high sACE levels (124). Even though the results of these HRCT scoring systems are promising, they are still not widely implemented in standard patient care.

### Fluor-18-Deoxyglucose Positron Emission Tomography

Over the past decade, ^18^F-FDG PET has gained territory as an important and reliable imaging biomarker in severe sarcoidosis (125).

Prior to ^18^F-FDG PET, gallium-67 (^67^Ga) scintigraphy was the most frequently used form of nuclear imaging in sarcoidosis, but ^18^F-FDG PET has replaced it, as it was shown to be more accurate in depicting extrathoracic lesions (126) and yields high quality images with superior contrast and spatial resolution compared to ^67^Ga scintigraphy (127). ^18^F-FDG PET can be used as a diagnostic marker, but also to predict treatment response and prognosis.

#### ^18^F-FDG PET as a Diagnostic Marker in Pulmonary Sarcoidosis

Although it is not necessary to obtain an ^18^F-FDG PET scan of every new sarcoidosis patient, it sometimes provides valuable information for the diagnostic process. In patients in whom the diagnosis of sarcoidosis is suspected, the extent of organ involvement and disease activity can be visualized using ^18^F-FDG PET. A prospective study of 36 newly diagnosed untreated sarcoidosis patients showed visual activity on ^18^F-FDG PET in 34 patients (94%). Furthermore, ^18^F-FDG PET was far more sensitive than ACE and sIL2R in this study (128).

In a small retrospective study of 23 biopsy-proven sarcoidosis patients with an indication to receive corticosteroid therapy, 91.7% had ^18^F-FDG PET positive findings. Sensitivity improved to 100% after skin involvement was excluded (129).

In a large retrospective study of 158 sarcoidosis patients, 75% had ^18^F-FDG PET positive findings (130). In some patients, new occult organ localizations such as cardiac or ossal sarcoidosis can be detected with ^18^F-FDG PET (131). As ^18^F-FDG PET can reveal occult localizations, it can also be used to determine the most suitable biopsy site (7). In a study of 89 patients with unexplained disabling symptoms, 73% had signs of disease activity on ^18^F-FDG PET, which were mostly extrathoracic (132).

#### ^18^F-FDG PET as a Marker of Therapeutic Response and Prognosis in Pulmonary Sarcoidosis

The natural disease course of sarcoidosis is very unpredictable, with not all patients being in need of systemic therapy. It is highly important, but very difficult, to distinguish patients who might benefit from treatment from those who will not.

**Figure 2:**
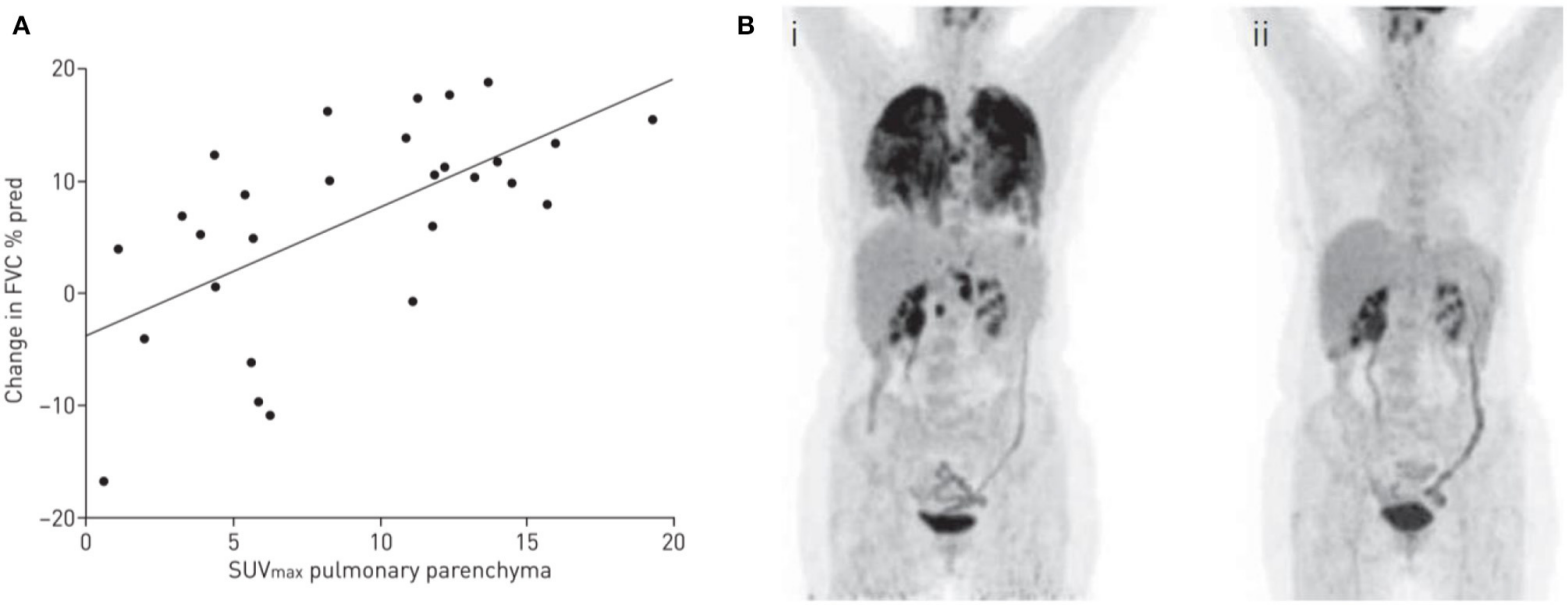
**(A)** ^18^F-fluorodeoxyglucose (FDG) by positron emission tomography (PET) activity and improvement in pulmonary function. Correlation between high activity of pulmonary parenchyma on ^18^F-FDG PET (maximum standardized uptake value [SUV_max_]) at baseline and improvement in forced vital capacity (FVC) in patients with a pulmonary treatment indication (*R* = 0.62, *p* = 0.0004). Reproduced and modified from (134) with permission. **(B)** Example of a PET-CT scan of a sarcoidosis patient with pulmonary involvement, before (i) and after (ii) 6 months of infliximab therapy.

A reasonable number of reports objectifying ^18^F-FDG PET as a predictor of treatment response have been published over the past few years. Keijsers et al. studied 43 newly-diagnosed sarcoidosis patients and found that patients with increased lung parenchymal activity on ^18^F-FDG PET had significantly lower DLCO. Moreover, high parenchymal activity was a predictor of improvement in pulmonary function upon treatment with corticosteroids. Patients without parenchymal activity assessed by ^18^F-FDG PET did not show decreasing pulmonary function when untreated (133). A prospective study included 56 severe sarcoidosis patients, refractory to first- and second-line treatment, who were treated with the anti-TNF drug infliximab. High ^18^F-FDG PET activity of the pulmonary parenchyma (SUVmax) at baseline was able to predict lung function improvement after 6 months of treatment (*R* = 0.62, *p* = 0.0004) (134) (Figure 2). As anti-TNF therapy is costly and known to cause side-effects, ^18^F-FDG PET can be very useful in therapeutic decision making, especially for this group of refractory patients.

In a study of 90 sarcoidosis patients with persistent symptoms, 72% had signs of activity on ^18^F-FDG PET. Furthermore, 38 patients (51%) with positive ^18^F-FDG PET results had normal ACE levels, suggesting an added value of ^18^F-FDG PET as an adjunct to serum biomarkers (135).

To date it is unknown what the optimal duration of systemic therapy should be. ^18^F-FDG PET is a potential tool to determine which patients are at risk for relapse of disease activity and symptoms. In a retrospective study, Vorselaars et al. (59) assessed the risk of relapse after discontinuation of infliximab therapy in severe sarcoidosis patients, and found that high activity (SUV_max_ > 6) of mediastinal lymphnodes on ^18^F-FDG PET is a predictor of relapse after discontinuation of infliximab therapy, with a hazard ratio of 4.33 (*p* < 0.001). This suggests that high activity on ^18^F-FDG PET reflects the burden of inflammation and therefore the need for prolonged therapy. A small retrospective study of 23 newly diagnosed sarcoidosis patients showed that persistent disease activity on ^18^F-FDG PET during the first months of corticosteroid treatment is a predictor of relapse (129).

Most studies have used the maximum standard uptake value (SUV_max_) to quantify disease activity. Recently, a new tool for the quantification of disease activity of the pulmonary parenchyma was proposed. Volumetric ^18^F-FDG PET analysis of global lung inflammation was proposed by Adams et al. (136) and possibly correlates better with conventional serum biomarkers ACE and sIL-2R than SUV_max_. TluG (Total Lung Glycolysis) measures the total activity of the pulmonary parenchyma, and although this derivative from the total lesion glycolysis in oncology studies looks promising, it was not superior to SUV_max_ in predicting lung function change during infliximab therapy (137).

### Cardiac Imaging Biomarkers

Current improved imaging techniques are leading to higher rates of incidence and recognition of cardiac sarcoidosis than previously reported (138). Approximately 5% of all sarcoidosis patients will have clinically relevant cardiac involvement (139).

#### Magnetic Resonance Imaging in Cardiac Sarcoidosis

The diagnosis of cardiac sarcoidosis can be challenging, as the diagnostic yield of endomyocardial biopsy is low and this procedure also has potential hazards (138). Cardiac Magnetic Resonance Imaging (CMR) is acknowledged in the diagnostic guidelines for cardiac sarcoidosis (140). Late gadolinium enhancement (LGE) on CMR (in a pattern consistent with cardiac sarcoidosis) in patients with known biopsy-proven sarcoidosis and no other explanation for cardiac disease can be used as a criterion to diagnose sarcoidosis (140).

Formation of edema or scar tissue in the myocardium or pericardium shows late gadolinium enhancement on CMR, as gadolinium is an extracellular contrast agent. Late gadolinium enhancement can potentially be optimized with the use of T1/T2 mapping, which can potentially discriminate between fibrosis (T1 mapping) and extracellular water/edema (T2 mapping) (141). T1/T2 mapping for this use needs further evaluation.

A large number of studies have found LGE on CMR to be an independent prognostic biomarker (142, 143). A large prospective cohort study of 321 sarcoidosis patients by Kouranos et al. (144) showed that LGE was an independent predictor of the primary endpoint (composite of death, life-threatening arrhythmias, admission for heart failure, and heart transplantation) with a hazard ratio of 5.7 (*P* < 0.004). Moreover, a large meta-analysis including 10 studies and a total of 760 patients concluded that patients with LGE on CMR were at increased risk of events, compared to patients without LGE (11.9 vs. 1.1%; *P* < 0.001), even if they had preserved left ventricle systolic function (11.6 vs. 0.7%; *P* < 0.0011) (145). A study investigating both LGE on CMR and uptake on ^18^F-FDG PET in 56 sarcoidosis patients with high clinical suspicion of cardiac sarcoidosis showed that LGE alone was the primary risk factor for events (arrhythmia/death). The authors suggest that future events may be driven by the presence of myocardial fibrosis instead of inflammation (146).

#### ^18^F-FDG PET in Cardiac Sarcoidosis

^18^F-FDG PET has an important role as a diagnostic marker for cardiac involvement and is included in the Hearth Rhythm Society Guideline for diagnosis of cardiac sarcoidosis. “Patchy uptake on dedicated cardiac PET (in a pattern consistent with Cardiac Sarcoidosis)” in the right context can point towards the diagnosis (140).

Due to the high rate of glucose metabolism in the human heart, cardiac assessment on ^18^F-FDG PET can be challenging. In a randomized trial of 82 patients, an 18-h fast with low carbohydrate diet preparation significantly reduced diffuse cardiac left ventricle uptake (147). A large meta-review including 559 patients revealed that the diagnostic sensitivity and specificity were significantly improved by increased fasting time and heparin administration before scanning (*P* = 0.01, 0.02) (148).

The added value of ^18^F-FDG PET in combination with CMR in the diagnostic process was studied in a cohort of 107 patients suspected of cardiac sarcoidosis. Added value was found in 45% of patients, as they were reclassified as likely to have cardiac sarcoidosis, most of them (80%) being correctly reclassified when compared with the final diagnosis (149).

Combining cardiac ^18^F-FDG PET with rubidium-82 to evaluate perfusion defects can increase the prognostic value. In a study including 118 consecutive cardiac sarcoidosis patients, the presence of both perfusion defects and increased FDG uptake identified patients at higher risk for VT or death HR 3.9 (*p* < 0.01) (150).

Newer PET/CMR scans are now available offering the possibility to perform cardiac ^18^F-FDG PET and CMR together, depicting both the pattern of injury and disease activity in a single scan (151).

Furthermore, ^18^F-FDG PET can be used to replace cardiac MRI in patients in whom the latter is contraindicated (e.g., devices, severe claustrophobia) (138).

### Neuroimaging Biomarkers

Neurosarcoidosis (NS) refers to the involvement of the central nervous system (CNS) and is seen in approximately 5% of all sarcoidosis patients. It is a serious and devastating complication which is difficult to diagnose because of the many different ways in which this disease can present itself (152, 153).

#### Magnetic Resonance Imaging in Neurosarcoidosis

The Zajicek criteria are commonly used to define the diagnosis of NS. In the Zajicek critera the diagnosis of NS is considered definite if there is biopsy confirmation of neural tissue, probable if there is evidence of neurological inflammation together with biopsy proven systemic sarcoidosis and possible when the presentation is typical and other potential causes have been excluded (154). In addition to histopathological tissue confirmation, imaging is used for the diagnosis of NS. Positive gallium-67 scintigraphy, MRI, ^18^F-FDG PET, and HRCT are used in different steps of the diagnostic process (155).

The gold standard to evaluate involvement of the CNS is the use of contrast enhanced MRI. The most common abnormalities seen in neurosarcoidosis patients at MRI are periventricular white matter lesions, meningitis or meningoencephalitis, solid parenchymal enhancing lesions, cranial neuritis, and hydrocephalus (156).

Unfortunately, NS is very hard to diagnose, especially in the absence of systemic sarcoidosis. In addition to a number of imaging methods the analysis of cerebrospinal fluid and blood are performed, despite the low sensitivity of these tests (157).

#### ^18^F-FDG PET in Neurosarcoidosis

As mentioned previously, MRI is the gold standard to evaluate CNS involvement in sarcoidosis. However, ^18^F-FDG PET can occasionally be helpful in demonstrating neurologic involvement not visualized on MRI (158). Furthermore, PET can help in detecting systemic manifestations of sarcoidosis other than neurological involvement (159) or assess disease activity during treatment of NS (160).

## Future Biomarkers

### JAK/STAT Signaling

The accumulation and activation of macrophages that form granulomas in sarcoidosis are driven by IFN-γ secretion. IFN-γ signaling is partly dependent on a specific signaling pathway named the Janus kinase/signaling transducer and activator of transcription (JAK/STAT) pathway (161). This pathway can activate a total of 6 STATs. Gene expression studies showed that the JAK/STAT signaling pathway is differentially expressed in most sarcoidosis patients, and that this pathway is more activated compared to healthy controls. Genes regulated by STAT1 in particular showed to be elevated in the blood of sarcoidosis patients (4, 162, 163). In addition to STAT1, STAT3 was also found to play a role in the granuloma formation. While STAT1 is primarily activated within granuloma macrophages, STAT3 is activated in between the granulomas in lymphocytes (162, 164).

Recently, various case reports have been published in which JAK/STAT inhibitors like tofacitinib and ruxolitinib were successfully used to treat patients with therapy-refractory sarcoidosis (165, 166).

It may be useful in the future to further unravel this pathway and focus on which specific patients may benefit from this therapy and whether JAK/STAT activation markers can act as biomarkers.

### mTOR Signaling

The mechanistic target of rapamycin (mTOR) signaling pathway is involved in cellular metabolism as well as proliferation and has been implicated in an increasing number of pathological conditions (167).

It has been demonstrated in a mouse model that granuloma can be formed spontaneously when the mTORC1-inhibitor TSC2 is depleted. It was also found that sarcoid granulomas from patients with active and progressive disease showed active mTORC1 signaling (168). A decreased expression of TSC1 was observed in 33% of the sarcoidosis patients studied, leading to increased mTORC1 activation (168). Interestingly, whole exome sequencing (WES) suggested that the mTOR signaling pathway could be involved in the development of familial sarcoidosis (169). In terms of clinical relevance, successful treatment of a sarcoidosis patient with the mTOR inhibitor rapamycin has been reported (133). As with JAK/STAT inhibition, it may also be useful in the future to further unravel the mTORC1 pathway in sarcoidosis, to see whether markers of mTORC1 activation can be used as therapeutic biomarkers in sarcoidosis. A focus of interest could be whether absence of mTORC1 activation can predict treatment response with rapamycin. Rapamycin has been successfully used to treat a patient suffering from sarcoidosis (89).

### Hair Cortisol

In sarcoidosis, 50–70% of the patients experience fatigue, some even experience chronic fatigue. Fatigue can still be present when sarcoidosis has clinically resolved, causing an impaired quality of life (170). One of the factors which is suspected to play a role in fatigue is stress. When the body is under psychological or physical stress, the hormone cortisol is released. Cortisol levels are commonly measured in serum, saliva, or urine, only acute changes are represented in this way. This could be overcome by measuring multiple samples during the day, but this would be labor-intensive and invasive, which in itself would increase cortisol release (171, 172). An emerging alternative technique is the analysis of scalp hair samples. Hair analysis has been done for decades, mostly to detect drugs of abuse. Hair samples are easy to collect in a non-invasive way and can be stored at room temperature.

The average scalp hair grows by 1 cm/month, so this method can retrospectively measure the production of these hormones over months (171, 172). The first study measuring cortisol levels of sarcoidosis patients in hair samples was performed by Van Manen et al. (171). This study showed that hair cortisol and cortisone levels were significantly higher in sarcoidosis patients compared to healthy controls, and that these increased levels related positively to the reported psychological distress, assessed by questionnaires. However, no differences in hair cortisol levels were found between sarcoidosis patients with and without fatigue.

Hair cortisol levels could be a future long-term biomarker for the measurement of chronic stress in sarcoidosis. A great advantage of this biomarker is that it is non-invasive and easily available, and can be used in a retrospective way. Using only one or a few hair strings, it may be possible to screen for fatigue and physical distress as well as to measure it in follow-up measurements. This can be used as an alternative to the currently used questionnaires, preventing variations in scores as to when patients fill in multiple questionnaires over time (171).

### Labeled PET-Tracers

A PET-traceable biomarker specific for sarcoid granulomas would expand the possibilities to diagnose this disease as well as response to treatment (127). ^18^F-FDG has been shown to be a useful PET-tracer in severe sarcoidosis. Other tracers, such as somatostatin receptors (SSTRs) like ^68^Ga-DOTA-NaI-octreotide (DOTANOC) or ^68^GA-DOTA-D-Phe-Tyr-octreotide (DOTATOC), would appear to be reliable alternatives to ^18^F-FDG. Multiple inflammatory cells that are found in granulomas, including epithelioid cells and macrophages, have SSTRs on their surface, while normal monocytes have not. In the case of cardiac sarcoidosis, DOTANOC is taken up by sarcoid lesions but not by normal myocardium (173). More research is needed to determine the clinical value of these different PET tracers.

Although PET is a useful technique, it might not be able to function as a diagnostic biomarker of sarcoidosis on its own. Nevertheless, this technique provides evidence supporting or refuting the diagnosis and gives additional information about active disease in specific organs.

Another interesting application is specific labeling of the target drug used in sarcoidosis treatment with PET tracers. Radioactively labeling infliximab with technetium-99 m (99mTC) allows responders to infliximab therapy to be distinguished from non-responders. This imaging method is already being used in several TNF-α mediated diseases, such as RA, inflammatory bowel disease (IBD) and spondyloarthropathy (174). In sarcoidosis, Vis et al. (174) quantified serum TNF-α levels with ^99m^TC-infliximab to evaluate disease activity and to identify responders, partial responders or non-responder priors to infliximab therapy. They showed that ^99m^TC-infliximab accumulation was highest in patients with an indication for systemic treatment (174). ^99m^TC-infliximab has not yet found its way into clinical practice for sarcoidosis.

## Omics

Sarcoidosis is a complex systemic disease reflecting immunological responses to different antigens in patients with certain genetic susceptibility (8, 175). Omics is an emerging field of research that encompasses genomics, epigenomics, transcriptomics, proteomics as well as metabolomics and is already widely used to understand polygenic and phenotypically diverse diseases and may also help identify more effective disease biomarkers in sarcoidosis (8, 175, 176).

In sarcoidosis, multiple diagnostic and prognostic biomarkers are already being identified by using single nucleotide polymorphism (SNP) technology, RNA sequencing and pathway analysis (175). Strong associations were found between sarcoidosis and various polymorphisms of the HLA allele and IL-1α. Various SNPs were also found to play a role in sarcoidosis pathogenesis, such as BTNL2, annexin A11, and NOTCH4, but the biological significance of several identified SNPs is unclear (noncoding) (175). A greater understanding of sarcoidosis may be obtained by moving beyond specific biomarkers to unbiased genome-wide analytic approaches. A study comparing lung tissue of sarcoidosis patients with that of healthy controls found that genes regulating macrophage-derived proteases and Th1 immune responses were differentially upregulated in sarcoidosis. Furthermore, IL23 and IL23R of the Th17 pathway and IL21 of STAT3 were also upregulated in sarcoidosis skin lesions compared to normal skin. Analyzing gene expression in peripheral blood proved to be a reliable surrogate for the use of tissue specimens, as it was able to reliably distinguish sarcoidosis patients from healthy controls, making this a useful approach to diagnosing and monitoring sarcoidosis (175). Based on current research, it is to be expected that the field of omics will further identify new promising biomarkers in sarcoidosis in the near future.

## Conclusion

Although numerous biomarkers have been evaluated for patients with sarcoidosis in recent decades, no gold standard has been set for their use in diagnosis or predicting disease course. Combinations of several serum biomarkers have been explored, with promising results, but the Holy Grail remains to be discovered. Future research should focus on combining serum or BALF biomarkers and further refined imaging techniques such as CT, MRI, and PET. Data on successful treatment of sarcoidosis patients with JAK/STAT and mTORC1 inhibitors suggests that these new immunological pathways should also be explored for new prognostic biomarkers in the near future.

## Author Contributions

RK, MJ, and AV wrote the manuscript, contributed to the concept, and designing of the work. AV and MV revised the work critically for important intellectual content. RK and MJ set up the reference database. All authors contributed to the article and approved the submitted version.

## Conflict of Interest

The authors declare that the research was conducted in the absence of any commercial or financial relationships that could be construed as a potential conflict of interest.
