# Supplementary material for: Biomarkers in the Diagnosis and Prognosis of Sarcoidosis: Current Use and Future Prospects
Source: Front Immunol. 2020 Jul 14;11:1443. doi: 10.3389/fimmu.2020.01443 (PMC7372102; doi:10.3389/fimmu.2020.01443)
Supplement: Supplementary file 1 [file Data_Sheet_1.PDF]

Table 1. Serum Biomarkers

Study	Inclusion	Subtype	Control group	Duration of follow-up	Change in biomarker	Sensitivity	Specificity	Correlation with Course
**ACE**								
Vorselaars 2015 (21)	(n=114) sarcoidosis patients started on second-line treatment with methotrexate. ACE was measured before and after methotrexate	(n=76) Patients with pulmonary treatment indication; (n=38) Patients with extrapulmonary treatment indication.		6 months	Mean ACE decreased from 71.4 U/L to 54.2 U/L			In patients with pulmonary treatment indication, ACE correlated with ΔVC, ΔFEV1, and ΔDLCO after methotrexate therapy
Kruit, 2007 (19)	(n=89) sarcoidosis patients	(n=51) Patients normalized or improved to stage I; (n=21) Patients with persistent stage II/III; (n=12) Patients developing fibrosis		Median duration of 10 years (range 1-27 years)	The DD group exhibited ACE levels that reached twice the value of that of the II group, whereas intermediate values were observed in the ID group.			ACE levels need to be correlated for I/D genotype. I/D allele frequencies can differ vastly between ethnicities.
Lopes, 2019 (20)	(n=46) sarcoidosis patients	(n=27) sarcoidosis patients had active disease and (n=19) patients were in remission		2 years	ACE concentrations were 341.18 ng/ml in control group, 337.866 ng/ml in sarcoidosis remission group and 470.96 ng/ml in active sarcoidosis group	88%	47%	Highest ACE levels were seen in patients at stages 0, 1, and 2.
Kahkouee, 2016 (13)	(n=148) Sarcoidosis patients diagnosed in accordance with ATS/ERS/WASOG criteria	Pulmonary sarcoidosis		3 years	There was a reverse correlation between chronicity and ACE level			Chronicity of sarcoidosis according to patients' HRCT presentations is inversely related to their ACE level
Lieberman, 1975 (14)	(n=28) Sarcoidosis patients with active disease		(n=200) Healthy controls		Serum ACE activity is both age and sex dependent; its activity is greater in men than in women of comparable age, and higher in those below 20 years of age			Determination of serum ACE activity should be useful for confirming a diagnosis of sarcoidosis and as a guide for determining the effectiveness of therapy.
Brice, 1995 (15)	(n=133) patients suffering from a variety of respiratory diseases	(n=51) sarcoidosis patients; (n=21) silicosis patients; (n=20) MTB patients; (n=12) cavitary tuberculosis patients; (n=26) COPD patients; (n=7) IPF patients.	(n=32) normal control volunteers		Significantly elevated levels of ACE were found in MTB, silicosis and sarcoidosis groups			Serum levels reflect the total granulomatous load
Studdy, 1978 (17)	(n=90) Sarcoidosis patients		(n=80) healthy controls		sarcoidosis patients who were not being treated with steroids had significantly			ACE assays provide useful information on the course of sarcoidosis and response to steroid treatment.

					higher ACE activity than treated patients			
Krasowski, 2015 (23)	(n=1292) patients with sACE levels measured between 2009 and 2015	(n=108) patients received ACE inhibitors		54 months	ACE levels measured in patients receiving ACEI therapy were substantially lower.			The use of ACE level to screen for sarcoidosis may result in missed or delayed diagnosis
**Lysozyme**								
Bergantini, 2019 (25)	(n=74) sarcoidosis patients	Persistent chronic disease		2 years	Lysozyme serum levels were elevated in 78% of sarcoidosis patients			Elevated lysozyme serum levels positively correlated with elevated KL-6 levels (r=0.35; p=0.004)
Tomita, 1999 (26)	(n=110) sarcoidosis patients from central Japan		(=30) Controls with other granulomatous diseases	5 years (n=41) sarcoidosis patients	In sarcoidosis patients mean serum lysozyme value was 15.7 ± 7.0 (μg/ml) while in controls mean serum lysozyme value was 11.0 ± 6.6 (μg/ml)	79.1%		The maximum serum lysozyme level had a tendency to increase significantly according to the number of organs involved
**Neopterin**								
Gelisgen, 2018 (28)	(n=59) sarcoidosis patients affected by chronic sarcoidosis	(n=20) Patients in active phase; (n=39) patients in inactive phase	(n=25) Healthy controls			88.1%	76%	While neopterin levels were increased in sarcoidosis patients in comparison to healthy controls, no differences were found between active and inactive disease
Ziegenhagen, 2003 (29)	(n=74) patients newly diagnosed with sarcoidosis	(n=10) Patients with Löfgren's syndrome; (n=51) with stable disease; (n=13) with active disease requiring treatment		6 months	Neopterin levels were slightly increased in sarcoidosis group			Neopterin levels were significantly increased in patients with active/progressive disease
**YKL40**								
Johansen, 2005 (33)	(n=27) Sarcoidosis patients with histological diagnosis		(n=173) healthy age-matched controls		Median serum YKL-40 levels were 420 μg/l (range 106–1000) in sarcoidosis patients and 100 μg/l (range 60–214 μg/l) in healthy controls			Patients with highest serum YKL-40 levels had lower DLCO/VA
Gelisgen, 2018 (28)	(n=59) Sarcoidosis patients affected by chronic sarcoidosis	(n=20) Patients in active phase; (n=39) in inactive phase	(n=25) Healthy controls			83.1%	92%	YKL-40 serum levels are elevated in patients with active sarcoidosis compared to inactive sarcoidosis
**sCD163**								
Tanimura, 2015 (36)	(n=40) Sarcoidosis patients and healthy controls	(n=20) Sarcoidosis patients	(n=20) Healthy controls		Mean sCD163 serum levels were significantly higher in sarcoidosis patients (1638 ± 134.8 ng/ml vs 550.6 ± 59.7 ng/ml in healthy controls)			
**CCL18**								

Cai, 2013 (82)	(n=199) patients with interstitial lung diseases	(n=54) IPF; (n=30) iNSIP; (n=11) RB-ILD/DIP; (n=26) COP; (n=34) HP; (n=44) sarcoidosis	(n=61) Healthy controls		CCL18 serum levels were significantly elevated in sarcoidosis patients: 108 ng/ml (IQR 67 ng/ml), in comparison to healthy controls 39 ng/ml (IQR 44 ng/ml)			No differences in CCL18 serum levels found between different radiological stages
Boot, 2010 (38)	(n=34) sarcoidosis patients with histologically proven sarcoidosis	(n=18) Patients with extended disease; (n=16) Patients with limited disease		16-25 months	CCL18 serum levels were elevated in sarcoidosis patients: median 259 ng/ml, range 71–1082 ng/ml; normal range 10–72 ng/ml	93%		
**CTO**								
Boot, 2010 (38)	(n=34) sarcoidosis patients with histologically proven sarcoidosis	(n=18) Patients with extended disease; (n=16) Patients with limited disease		16-25 months	CTO serum levels were elevated in all sarcoidosis patients: median 577 nmol/ml h, range 74–3032 nmol/ml h; normal median 42 nmol/ml h, range <70 nmol/ml h	100%		
Lopes, 2019 (20)	(n=46) sarcoidosis patients	(n=27) sarcoidosis patients with active disease; (n=19) patients in remission		2 years	Mean serum CTO activities were 65.55 U/ml in control group, 38.096 U/ml in sarcoidosis remission group and 297.11 U/ml in active sarcoidosis group	55%	100%	mean CTO activity was higher in patients with active disease than in controls or patients in remission
**sIL-2R**								
Vorselaars, 2015 (21)	(n=114) sarcoidosis patients started on second-line treatment with methotrexate. sACE was measured before and after methotrexate	(n=76) patients with pulmonary treatment indication; (n=38) patients with extrapulmonary treatment indication.		6 months	Mean sIL-2R decreased from 4840 pg/ml to 2290 pg/ml			Significant correlation was found between ΔsIL-2R and ΔFVC, ΔDLCO after methotrexate therapy in patients with a pulmonary treatment indication
Grutters, 2003 (61)	(n=14) Diagnosis was confirmed by histologic evidence.	All patients had active disease		2 years				Positive correlation between change in sIL-2R and change in radiographic stage, which remained significant after correction for treatment. The initial sIL-2R level correlated inversely with the extent of change in the follow-up sIL-3r level
Ogata-suetsugu, 2017 (83)	(n=67) newly diagnosed sarcoidosis patients	(n=52) Patients were observed by chest radiography		37 months	Serum sIL-2R values were elevated in 45.9% of sarcoidosis patients. Mean 818.8 ± 453.1			Serum sIL-2R levels in patients with pulmonary scadding stages II and III were significantly higher than stage 0 and I

					U/ml (normal <206 U/ml)			
Vorselaars, 2014 (59)	(n=47) sarcoidosis patients who started infliximab therapy			36.6 ± 22.6 months	Serum sIL-2R concentrations of ≥4000 pg·mL^{-1} at the start of therapy were found to predict relapse			
**BAFF**								
Ando, 2018 (64)	(n=88) sarcoidosis patients	Pulmonary sarcoidosis	(n=21) Healthy controls		Median BAFF serum levels in sarcoidosis patients were elevated (1553.0 pg/ml, range 535.0-7958.0) in comparison to healthy controls (984.6 pg/ml, range 585.0-1234.1)			Serum BAFF levels show a positive correlation with extrapulmonary involvement
Saussine, 2012 (65)	(n=33) sarcoidosis patients	(n=18) Patients with active sarcoidosis; (n=15) patients with inactive sarcoidosis	(n=18) Healthy controls		BAFF serum levels in patients with active sarcoidosis (2343 ± 1079 pg/ml) were significantly higher than in patients with inactive sarcoidosis (1239 ± 376 pg/ml) and healthy controls (1352 ± 526 pg/ml).			
Ueda-Hayakawa, 2013 (66)	(n=37) sarcoidosis patients		(n=21) Healthy controls		Serum BAFF levels were significantly higher in sarcoidosis patients (1.59 ± 0.92 ng/ml) than in healthy controls (0.97 ± 0.21 ng/ml)			The frequency of skin and eye involvement was significantly higher in patients with elevated BAFF serum levels
**B-cells**								
Lee, 2011 (69)	(n=22) sarcoidosis patients with biopsy-proven sarcoidosis	Patients with severe chronic sarcoidosis			Memory B-cells were significantly reduced in sarcoidosis patients			
Kudryavtsev, 2020 (62)	(n=37) sarcoidosis patients with stage II sarcoidosis	Pulmonary sarcoidosis	(n=35) Sex- and age-matched healthy controls		The frequency of CD19+ B-cells was higher in sarcoidosis patients, memory B-cells were decreased and activated naïve B-cells were increased in sarcoidosis patients			
Kamphuis, 2013 (70)	(n=32) sarcoidosis patients				CD271 memory B-cells were significantly reduced in sarcoidosis patients while total B-cell			

					numbers were not affected			
**Bregs**								
Saussine, 2012 (65)	(n=33) sarcoidosis patients	(n=18) Patients with active sarcoidosis; (n=15) patients with inactive sarcoidosis	(n=18) Healthy controls		Patients with active sarcoidosis had increased numbers of IL-10 producing Bregs compared to inactive disease and healthy controls			
Kudryavtsev, 2020 (62)	(n=37) sarcoidosis patients with stage II sarcoidosis	Pulmonary sarcoidosis	(n=35) Sex- and age-matched healthy controls		Sarcoidosis patients have increased percentages of regulatory B-cells			
