# Supplementary material for: Biomarkers in the Diagnosis and Prognosis of Sarcoidosis: Current Use and Future Prospects
Source: Front Immunol. 2020 Jul 14;11:1443. doi: 10.3389/fimmu.2020.01443 (PMC7372102; doi:10.3389/fimmu.2020.01443)
Supplement: Supplementary file 2 [file Data_Sheet_2.PDF]

Table 2. BALF Biomarkers and cytokines

Study	Inclusion	Subtype	Control group	Duration of follow-up	Change in biomarker	Sensitivity	Specificity	Correlation with Course
**Lymphocytosis**								
Hyldgaard, 2012 (80)	(n=18) Biopsy proven sarcoidosis patients		(n=73) Patients with other pulmonary diseases	2 years	Elevated lymphocyte percentages in BALF in sarcoidosis patients compared to other pulmonary diseases	71%	68%	BAL lymphocytosis is not a universal finding in sarcoidosis but proved useful for the diagnosis
Tanriverdi, 2015 (79)	(n=68) Biopsy proven sarcoidosis patients.	Sarcoidosis patients with diffuse parenchymal lung disease	(n=72) non-sarcoidosis patients with diffuse parenchymal lung disease: (n=20) CTD-ILD; (n=14) pneumoconiosis; (n=12) IPF; (n=5) infections; (n=16) other ILD; (n=4) malignancy	10 years, retrospectively	BAL lymphocyte fraction was significantly higher in sarcoidosis than in non-sarcoidosis ILDs (20.6% to 6%)	85%	72%	BAL lymphocyte fraction and CD4/CD8 ratio were 2.5 - 3 fold greater in sarcoidosis than in non-sarcoidosis ILD patients
**CD4/CD8 ratio**								
Ziegenhagen, 2003 (29)	(n=74) Sarcoidosis patients diagnosed in accordance with ATS/ERS/WASOG criteria	Pulmonary sarcoidosis	(n=48) Individuals who underwent bronchoscopy for diagnostic reasons and were retrospectively free of any infectious, inflammatory or malignant lung disease.	6 months	The CD4/CD8 ratio was significantly increased in the sarcoidosis group compared to controls.			Although the BALF CD4/CD8 ratio may be useful in establishing a diagnosis of sarcoidosis, it did not reflect the severity of the disease
Hamsten, 2016 (78)	(n=251) Sarcoidosis patients with active disease, diagnosis based on ATS/ERS/WASOG criteria.	Pulmonary sarcoidosis	(n=16) Healthy controls (never-smokers) with no respiratory infections					For CD4/CD8 ratio no associations were observed between patients and controls
Tanriverdi, 2015 (79)	(n=68) Biopsy proven sarcoidosis patients.	Sarcoidosis patients with diffuse parenchymal lung disease	(n=72) non-sarcoidosis patients with diffuse parenchymal lung disease: (n=20) CTD-ILD; (n=14) pneumoconiosis; (n=12) IPF; (n=5) infections; (n=16) other ILD; (n=4) malignancy		Median CD4/CD8 ratio in BALF was significantly higher in sarcoidosis than in non-sarcoidosis ILDs (3.87 to 0.88)	76%	79%	BAL lymphocyte fraction and CD4/CD8 ratio were 2.5 - 3 fold greater in sarcoidosis than in non-sarcoidosis ILD patients
Hyldgaard, 2012 (80)	(n=18) Biopsy proven sarcoidosis patients		(n=73) Patients with other pulmonary diseases	2 years	Significantly elevated CD4/CD8 ratio in sarcoidosis patients compared to other pulmonary diseases	68%	73%	The diagnosis of sarcoidosis is supported by an elevated CD4/CD8 ratio
**CD103+CD4+/CD4+ ratio**								
Kolopp-Sarda, 2000 (84)	(n=18) patients with $CD4^+$ lymphoc		(n=16) patients with $CD4^+$ lymphocyt		The percentage of CD103			

	ytosis and confirmed pulmonary sarcoidosis according to clinical, radiological, and pathology criteria		osis without sarcoidosis, (n=48) Patients without $CD4^+$ lymphocytosis, (n=11) patients with normal BAL outcome (lymphocytes < 10%, polymorphonuclear cells < 5%)		expressing CD4+T-cells were significantly lower in the sarcoidosis group (9.5 ± 1.4%) compared to all other groups (33.8 ± 17.1%)			
Heron, 2008 (85)	(n=55) Biopsy proven sarcoidosis patients		(n=1) Löfgren's syndrome, (n=22) Hypersensitivity pneumonitis, (n=8) IPF, (n=3) Other interstitial pneumonia, (n=13) Infection, (n=4) TBC, (n=8) Systemic disease, (n=6) Malignancy, (n=1) Non-hodgkin lymphoma, (n=1) multiple myeloma, (n=1) chronic lymphomatic leukemia, (n=3) other.		Sarcoidosis patients showed a significantly lower CD103+CD4+/CD4+ ratio compared to patients with other ILDs (0.16±0.02 vs. 0.4± 0.03).	57%	91%	
Bretagne, 2016 (86)	(n=53) Sarcoidosis patients with confirmed diagnosis	(n=22) by tissue biopsy, (n=25) by transbronchial needle aspiration or meadiastinal lymph nodes, (n=6) typical Löfgrens syndrome.	(n=22) Tuberculosis, (n=36) other pulmonary infections, (n=6) HP, (n=14) NSIP, (n=9) OP, (n=19) drug-induced pneumonia, (n=21) other ILDs, (n=25) other diagnosis.	2.3 years	The CD103+CD4+/CD4+ ratio was significantly lower in sarcoidosis compared to NSIP, HP and other ILDs. However, its discriminative power to distinguish between sarcoidosis and all other diagnoses was low			
Hyldengaard, 2012 (80)	(n=19) Sarcoidosis patients with biopsy confirmed diagnosis	(n=11) Scadding stage 0/I, (n=8) scadding stage II/III	(n=88) other pulmonary diseases, (n=12) EAA, (n=10) IPF, (n= 6) NSIP, (n=2) desquamative interstitial pneumonitis, (n=1) lymphocytic interstitial pneumonitis, (n=10) collagen vascular disease with interstitial lung disease, (n=26) unclassified interstitial lung disease, (n=2) tuberculosis, (n=1) aspergillosis and (n=18) other		No significant differences were detected in CD103+CD4+/CD4+ in BAL fluid between the patient groups	35%	93%	

			non-granulomatous lung diseases					
**CD4+ Vα2.3+ T-cells**								
Darlington, 2020 (83)	(n=749) Sarcoidosis patients diagnosed in accordance with WASOG criteria	(n=274) patients with Löfgren's syndrome; (n=475) non-LS patients	(n=69) healthy volunteers; (n=39) patients with other pulmonary conditions		An increased proportion of CD4+ Vα2.3+ T-cells in BALF is highly specific for sarcoidosis	97%	36%	This T-cell subset could be used in addition to the CD4/CD8 ratio to support the sarcoidosis diagnosis, especially in LS
**Th17-cells**								
Facco, 2010 (87)	(n=25) Biopsy proven sarcoidosis patients	Patients with pulmonary involvement	(n=10) Healthy controls	6 months	High expression of Th17 (CCR6) in sarcoidosis patients			Th17 cells infiltrate sarcoid lung, localizing around and inside the granuloma, at the sites of disease activity, not only in the early phase, but also in the progression towards the fibrotic phase of the disease
Ramstein, 2016 (89)	(n=65) sarcoidosis patients	(n=35) Sarcoidosis patients from U.S. cohort; (n=30) sarcoidosis patients from Erasmus cohort	(n=18) Healthy controls from U.S. Cohort; (n=12) healthy controls from Erasmus Cohort		Numbers of Th17 cells were significantly increased in sarcoidosis patients compared to healthy controls			
Broos, 2018 (3)	(n=55) Sarcoidosis patients donated BALF, mediastinal lymph nodes (MLNs)-derived fine needle aspiration or peripheral blood		(n=22) Lung transplantation donors without signs of pulmonary inflammation		In sarcoidosis BALF, Th17.1 cell proportions were higher than either Th1, Th2, Th17 or CCR6+ double-positive cells			Th17 levels are highest in BALF compared to MLN
**Treg**								
Kachamakova - Trojanowska, 2018 (92)	(n=45) newly diagnosed treatment-naive sarcoidosis patients	patients with pulmonary stage I and II	(n=35) Healthy controls		Patients with pulmonary sarcoidosis have a high percentage of Tregs			
**Neutrophils**								
Tutor-Utora, 2006 (97)	(n=33) Non-smoking sarcoidosis patients diagnosed in accordance with ATS/ERS/WASOG criteria	Pulmonary sarcoidosis		31 months	Higher percentage of neutrophils in sarcoidosis patients with advanced disease			Patients with a poor outcome had a significantly higher percentage of neutrophils
Ziegenhagen, 2003 (29)	(n=74) Sarcoidosis patients diagnosed in accordance with ATS/ERS/WASOG criteria	Pulmonary sarcoidosis	(n=48) Individuals who underwent bronchoscopy for diagnostic reasons and were free of any inflammatory or malignant lung disease.	6 months	A significant increase in the % BALF neutrophils in sarcoid patients with progressing disease compared to controls			The percentage of neutrophils in BALF is significantly elevated in sarcoidosis patients requiring systemic steroid therapy.
**NK-cells**								
Tutor-utora, 2006 (97)	(n=33) Non-smoking	Pulmonary sarcoidosis		31 months	NK-cells in BALF were			Patients with a poor outcome

	sarcoidosis patients diagnosed in accordance with ATS/ERS/WASOG criteria				lower in patients at Stage I than in patients at Stage II and III			and in need of corticosteroid treatment had a higher percentage of NK-cells
Bergantini, 2019 (98)	(n=190) patients with sarcoidosis and other ILDs from 2015 to 2018	(n=115) sarcoidosis patinets	(n=24) cHP patients; (n=32) IPF; (n=11) NSIP patients; (n=8) controls	3 years	Lower NK-cell percentages were observed in sarcoidosis patients than in the other ILD groups (IPF and NSIP) (p<0.05)	46%	66%	Chronic granulomatous lung disorders (sarcoidosis and cHP) showed median NK percentages of <1.8%, in contrast with controls 2.4% and severe ILD (IPF and NSIP) 3%
**NKT-cells**								
Bergantini, 2019 (98)	(n=190) patients with sarcoidosis and other ILDs from 2015 to 2018	(n=115) sarcoidosis patinets	(n=24) cHP patients; (n=32) IPF; (n=11) NSIP patients; (n=8) controls	3 years	Lower NKT-cell percentages were observed in sarcoidosis patients than in the other ILD groups (IPF and NSIP) (p<0.05)	70%	56%	Chronic granulomatous lung disorders (sarcoidosis and cHP) showed median percentages of NKT-cells of 4.8%, 5.7% in controls and 6.2% in other ILDs (IPF and NSIP).
Kobayashi, 2004 (101)	(n=43) sarcoidosis patients with no history of using steroids or other anti-inflammatory drugs	(n=30) Remitting; (n=13) non-remitting.	(n=22) Normal controls		Significantly lower levels of IFN-y producing NKT-cells in patients with non-remitting sarcoidosis compared to those with remitting disease and control samples			Dysfunction of NKT-cells contribute to the modulation of disease progression and the formation of non-caseating granulomas in sarcoidosis
Kobayashi, 2004 (101)	(n=43) sarcoidosis patients with no history of using steroids or other anti-inflammatory drugs	(n=30) Remitting; (n=13) non-remitting.	(n=22) Normal controls		Significantly higher number of NKT-cells among total T-cells in lymph nodes of sarcoidosis patients.			Sarcoidosis involves an accumulation of NKT cells in granuloma lesions
Korosec, 2010 (102)	(n=47) newly diagnosed, histologically confirmed sarcoidosis according to ATS/ERS/WASOG criteria	Pulmonary sarcoidosis; (n=14) Lofgren patients; (n=15) Stage 1; (n=28) stage II; (n=3) stage III (n=1) stage IV	(n=8) control subjects without any pulmonary morbidities		Frequency of NKT cells were comparably low in both clinical categories of sarcoidosis patients; Lofgren's syndrome vs. other forms; 0.21% vs. 0.19%			There is a major pulmonary deficiency of NKT-cells in the lungs of newly identified, corticosteroid-naïve sarcoidosis patients, which is approximately 5 times lower compared to control subjects
**CXCL9, CXCL10, CXCL11**								
Arger, 2020 (106)	(n=108) Sarcoidosis patients who met criteria established by ATS				Higher CXCL9 and CXCL10 levels in BALF, lung tissue and blood of sarcoidosis patients			Higher levels of CXCL10 were associated with lower FVC, TLC and DLCO values while higher CXCL9 levels were associated with

								involvement of multiple organs
Arger, 2019 (107)	(n=104) Sarcoidosis subjects who met established ATS diagnostic criteria		(n=49) Healthy controls from the Bay Area community	5 years	CXCL11 is increased in BALF, alveolar macrophages and lung tissue in sarcoidosis patients			CXCL11 levels were significantly increased in subjects with abnormal FVC, FEV1, and DLCO
Busuttil, 2009 (105)	(n=72) Sarcoidosis patients staged according to ATS/ERS/WASOG criteria.	Pulmonary sarcoidosis			In sarcoidosis, CXCL9 levels were elevated compared to healthy controls			Sarcoid granulomas are made up of CXCR3-expressing lymphocytes and cells of monocyte lineage
**KL-6**								
Kunitake, 2001 (113)	(n=41) sarcoidosis patients with biopsy evidence		(n=13) Healty controls		KL-6 levels were elevated in BALF of sarcoidosis patients 280 ± 212 U/ml in comparison to healthy controls 137 ±61 U/ml			
Janssen, 2003 (114)	(n=79) Sarcoidosis patients with PA	(n=56) non-smokers, (n=23) smokers	(n=38) Healthy controls	2 years		86%	84%	KL-6 levels were highest in stage II/III patients and/or patients with parenchymal infiltration
Bergantini, 2019 (25)	(n=74) sarcoidosis patients	Persistent chronic disease		2 years	KL-6 levels were elevated in sarcoidosis patients (573±480 IU/ml) in comparison to healthy controls (267.7±147.7 IU/ml)	78%	73%	Elevated lysozyme serum levels positively correlated with elevated KL-6 levels (r=0.35; p=0.004)
d'Alessandro, 2020 (115)	(n=100) Caucasion patients with ILD of suspected ILD	(n=41) Pulmonary sarcoidosis patients, (n=11 cHP) patients, (n=24) other ILD	(n=24) patients with no ILD		KL-6 levels were significantly higher in sarcoidosis patients than in other ILD	73%	69%	Elevated KL-6 levels correlated with CD4+/CD8+ ratio
Myioshi, 2010 (77)	(n=43) Pulmonary sarcoidosis patients diagnosed according to ATS/ERS/WASOG criteria			2 years	Serum KL-6 levels significantly elevated in sarcoidosis patients with parenchymal infiltration	76.9%	70%	KL-6 levels were predictive of increased parenchymal infiltration.
